# Predicting gene expression using millions of yeast promoters reveals *cis*-regulatory logic

**DOI:** 10.1093/bioadv/vbaf130

**Published:** 2025-06-02

**Authors:** Tirtharaj Dash, Susanne Bornelöv

**Affiliations:** Cancer Research UK Cambridge Institute, University of Cambridge, Li Ka Shing Centre, Cambridge CB2 0RE, United Kingdom; Department of Biochemistry, University of Cambridge, Cambridge CB2 1GA, United Kingdom; Cancer Research UK Cambridge Institute, University of Cambridge, Li Ka Shing Centre, Cambridge CB2 0RE, United Kingdom; Department of Biochemistry, University of Cambridge, Cambridge CB2 1GA, United Kingdom

## Abstract

**Motivation:**

Gene regulation involves complex interactions between transcription factors. While early attempts to predict gene expression were trained using naturally occurring promoters, gigantic parallel reporter assays have vastly expanded potential training data. Despite this, it is still unclear how to best use deep learning to study gene regulation. Here, we investigate the association between promoters and expression using Camformer, a residual convolutional neural network that ranked fourth in the Random Promoter DREAM Challenge 2022. We present the original model trained on 6.7 million sequences and investigate 270 alternative models to find determinants of model performance. Finally, we use explainable AI to uncover regulatory signals.

**Results:**

Camformer accurately decodes the association between promoters and gene expression ($r^{2}=0.914\pm 0.003$, ρ=0.962 ± 0.002) and provides a substantial improvement over previous state of the art. Using Grad-CAM and in silico mutagenesis, we demonstrate that our model learns both individual motifs and their hierarchy. For example, while an IME1 motif on its own increases gene expression, a co-occurring UME6 motif instead strongly reduces gene expression. Thus, deep learning models such as Camformer can provide detailed insights into *cis*-regulatory logic.

**Availability and implementation:**

Data and code are available at: https://github.com/Bornelov-lab/Camformer.

## 1 Introduction

Gene regulation is a fundamental aspect of life, in which cells control when and to what extent specific genes are activated or repressed. The gene-regulatory process is tightly controlled and dynamically adjusted in response to internal and external signals. Many such signals reside in the gene promoter region, in the form of short sequence motifs that are recognized and bound by transcription factors (TFs). However, such TF motifs, or near-matching motifs, are frequent, and their effect on gene expression depends on the surrounding sequence context, including competition and cooperation between different TFs. Despite considerable efforts to map the gene-regulatory network through the characterization of individual TFs (Gerstein *et al.* 2012, Guo *et al.* 2012), fully decoding the *cis*-regulatory code remains an open challenge. One key limitation has been the lack of suitable data to study TF cooperativity and spacing rules, as the genome itself does not contain enough TF motif pairs to draw statistically robust conclusions (Avsec *et al.* 2021a).

Recently, deep learning approaches, fuelled by the increasing availability of large-scale genomic data, have emerged as a promising technique for studying gene regulation. DeepSEA (Zhou and Troyanskaya 2015) pioneered the use of convolutional neural networks (CNNs) to identify regulatory single-nucleotide variants (SNVs) by predicting their effects on 166 chromatin-binding factors. DanQ (Quang and Xie 2016) demonstrated that predictive performance was improved by integrating a bi-directional long short-term memory (LSTM), which was hypothesized to capture motif interdependencies. DeepAtt (Li *et al.* 2020) introduced category attention, which reduced model size while further improving performance. Beyond regulatory variants, Zrimec *et al.* (2020) trained a model on 20 000 mRNA datasets, revealing how mRNA and flanking DNA sequences interact and associate with gene expression. BPNet (Avsec *et al.* 2021a) employed a CNN to provide base-resolution insights into binding and cooperativity of four TFs. Finally, Enformer (Avsec *et al.* 2021b) used a combination of convolutional and transformer blocks to handle longer sequences, enabling it to predict long-range interactions between enhancers and promoters.

These earlier models were trained on naturally occurring genomic sequences, which imposes a strong upper limit on the size and diversity of training data. However, massively parallel reporter assays (MPRAs) enable sequencing-based readouts of the activities of thousands of candidate regulatory elements in a single experiment. Traditionally, such assays used a predefined set of sequences. By using random sequences, gigantic parallel reporter assays (GPRA) have effectively enabled an unlimited amount of training data (de Boer *et al.* 2020). Conducting a GPRA, de Boer *et al.* (2020) constructed a library of >100 million (M) random promoters and measured their ability to drive the expression of yellow fluorescent protein (YFP) in yeast (*Saccharomyces cerevisiae*). This study, along with a follow-up study (Vaishnav *et al.* 2022), provides valuable benchmarking data, facilitating the development and comparison of deep learning methods for studying the *cis*-regulatory code.

Community-wide challenges and systematic benchmarking can drive the development of computational methods. For instance, AlphaFold (Jumper *et al.* 2021) popularized protein structure prediction by winning the Critical Assessment of Structure Prediction (CASP) competition in 2018. Similarly, the Dialogue for Reverse Engineering Assessment and Methods (DREAM) competition aims to improve our understanding of biological processes by advancing bioinformatic methods (Meyer and Saez-Rodriguez 2021). The Random Promoter DREAM challenge held in 2022 (DREAM2022) specifically sought to advance deep learning methods for genomics. The participants were provided with GPRA data representing 6 739 258 promoters and their YFP expression levels. The goal of the challenge was to predict the expression of another 71 103 promoters, which were withheld during the competition. In total, 19 teams outperformed a transformer model, which represented previous state of the art (SOTA) (Vaishnav *et al.* 2022, Rafi *et al.* 2024). Among these top models were LegNet (Penzar *et al.* 2023) in 1st place, Proformer (Kwak *et al.* 2024) in 3rd place, and CRMnet (Ding *et al.* 2023) in 12th place.

Camformer secured fourth place by designing a CNN with residual connections. Surprisingly, although we originally attempted to build a transformer model, all our best-performing models were CNNs. Here, we provide an in-depth analysis of the submitted model, including a systematic grid search to pinpoint factors influencing model performance. Additionally, model explainability techniques revealed both known and potentially novel gene-regulatory motifs and interactions. Overall, our study demonstrates that a carefully designed CNN can achieve SOTA performance on gene-regulatory tasks, while providing important insights into the *cis*-regulatory code.

## 2 Materials and methods

### 2.1 Training data and evaluation

To simultaneously profile millions of promoters for their effect on gene expression, Rafi *et al.* (2024) conducted a GPRA. A library of 80-nucleotide (nt) random DNA sequences was cloned into a minimal promoter construct located upstream of a YFP and followed by a constitutively expressed red fluorescent protein (RFP). The resulting plasmid was transformed into yeast, its promoter activity was measured as by flow cytometry log(YFP/RFP), and the cells were sorted into 18 expression bins that were sequenced separately. An expression value was assigned to each promoter, describing its weighted mean expression bin.

**Figure 1.**
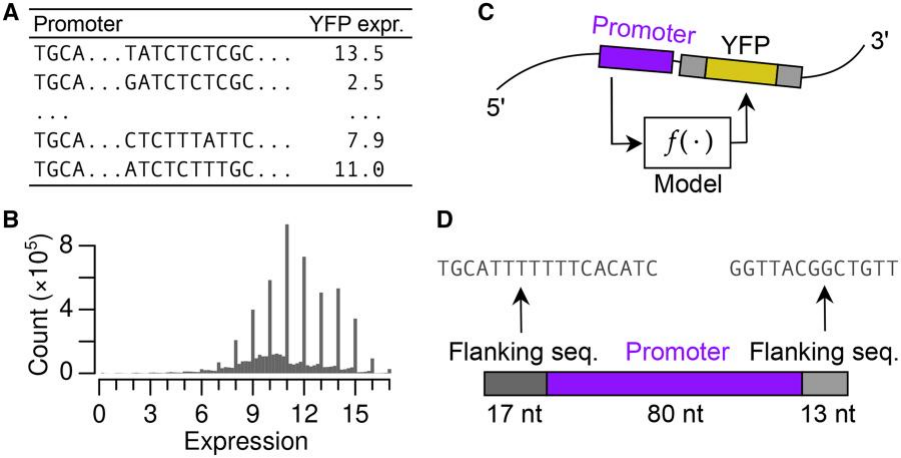
Schematic of prediction task: (A) training data are arranged as (promoter, expression) pairs, (B) histogram of gene expression in training set, (C) the task is to predict YFP expression given a promoter sequence, and (D) promoter sequences are composed of an 80 nt random sequence positioned between two fixed sequences.

In total, the public training set consisted of 6 739 258 promoter sequences along with their expression (mean of ∼2 cells per promoter) (Fig. 1A and B). A test set with 71 103 promoter sequences was sequenced separately, at higher depth, enabling a more precise estimation of their expression (mean of ≥100 cells per promoter). Furthermore, the test included promoter sequences with different properties: sequences with SNVs (*n* = 44 340 pairs), perturbation of known TF binding sites (n=3287 pairs), tiling of TF binding sites across random sequences (n=2624 pairs), sequences with high (n=968) and low (n=997) expression levels, native yeast promoters (n=997), challenging sequences (n=1953), and random sequences (n=6349). The test set was kept private during the competition, with the exception of 13% that was used for the public leaderboard, where only $r^{2}$, ρ, and weighted Pearson and Spearman scores were shown.

### 2.2 Prediction task

The prediction task can be defined as follows. Our study explores how to define *f* using a CNN (Fig. 1C).

Definition 1. Let $S={\left\{A,C,G,T,N\right\}}^{110}$ denote a promoter sequence of length 110. Here, *A*, *C*, *G*, and *T* are the four nucleotides and *N* represents an unknown nucleotide. The gene expression prediction task is then to learn a mapping f:S→R.

### 2.3 Data preprocessing

Each promoter was represented by its ∼80 nt variable region inserted between two fixed sequences of length 17 and 13 nts, respectively, representing the flanking plasmid sequence (Fig. 1D). We used only sequences of length 110±3 nts (discarding 110 138 sequences) and containing at most three *N* (discarding 23 533 sequences). Sequences <110 nts were padded with *N* at the end, and sequences >110 nts were truncated.

## 3 Algorithm and implementation

Although transformers are currently trending in sequence learning (Lin *et al.* 2022), there have been numerous successful CNNs for genomics. Here, we investigate whether a carefully designed CNN can achieve SOTA performance in predicting gene expression from promoter sequences. We base this on the inherent ability of a CNN to extract sequence motifs from DNA sequences. If each kernel is seen as a position weight matrix (PWM), then each convolutional layer corresponds to calculating the PWM score across the input sequence.

Details on the hardware used for training are provided in Methods 1.1, available as supplementary data at *Bioinformatics Advances* online.

### 3.1 Submission model

We first sought to determine an optimal structure for the CNN. There are two ways to do this: (i) a classical grid search over different configurations or (ii) using an automated Bayesian search. As a Bayesian search allows exploration of a larger search space, this approach was used for the competition. Nevertheless, to gain a better insight into model design choices, we later performed systematic experiments using a grid search and elaborate on this in the subsequent sections.

#### 3.1.1 Sequence encoding

Preprocessed training (*n *= 6 605 587) and test (*n *= 71 103) sequences were one-hot encoded using: A=[1,0,0,0], C=[0,1,0,0], G=[0,0,1,0], T=[0,0,0,1], and the special symbol N=[0,0,0,0]. Hence, each promoter sequence is uniformly represented as a tensor of dimension (height[h],width[w],channels[c])=(110,1,4).

#### 3.1.2 Training loss function

Since training data were expected to be noisy, we used mean absolute error (MAE), also called $L_{1}$ loss, between true and predicted expression as the loss function. This allows the outliers to be handled in a more robust manner.

#### 3.1.3 Optimizer

We used the Adam optimizer with weight decay regularization (AdamW). Maximum number of epochs was set to 50, but in practice, we used early stopping to obtain a well-generalized model by monitoring performance (r+ρ) on a validation set (8% of the training data).

#### 3.1.4 Model refinement

Candidate models were construed as described in Methods 1.2, available as supplementary data at *Bioinformatics Advances* online and refined using a the Optuna hyperparameter optimization software (Akiba *et al.* 2019). We varied batch size, learning rate, weight decay, size of the convolutional kernels, number of convolutional and fully connected layers, and number of channels and dropout rates. Most runs tested up to 100 models across 1 or 2 M promoters. The most promising models were then re-trained on the full training set. We noticed that several independent runs converged towards adding a single max pooling operation at the penultimate layer. Indeed, although our final model did not originally include such pooling operation, adding it manually improved its performance.

#### 3.1.5 Camformer

**Figure 2.**
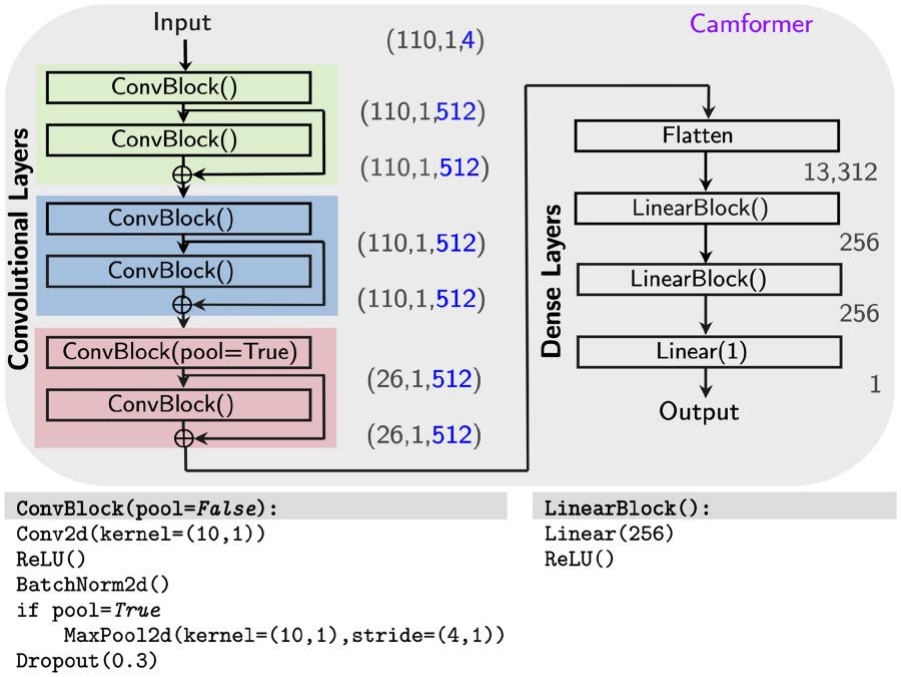
The final model consists of six convolutional layers followed by fully connected layers. Inputs and activations are shown in the format (h,w,c).

The model submitted for DREAM2022 is shown in Fig. 2B and referred to as ‘Camformer’. The final model has six convolutional layers with residual connections after layers 2, 4, and 6, followed by three fully connected layers to compute the predicted expression. Overall, the model had 16.6 M trainable parameters.

### 3.2 Hyperparameter grid search

To determine what factors contributed to model performance, we performed a systematic grid search over model structures, different sequence encoding strategies, loss functions, optimizers, and learning rate schedulers. Due to the systematic evaluation performed, this experiment allowed us to probe the effect of each of the tested variables. To keep the experiment tractable, we restricted ourselves to three basic structures of Camformer: Camformer, Camformer-Small, and Camformer-Mini. These models and the full grid search are described in Methods 1.3, available as supplementary data at *Bioinformatics Advances* online.

### 3.3 Evaluation

#### 3.3.1 Quantitative, predictive evaluation

All final models were trained using the training set (with 10% of the data used as a validation set for early stopping) and evaluated on the full external test set. We adopt the Pearson and Spearman correlation coefficients (*r* and ρ, respectively) between true and predicted expressions for evaluating the models. Evaluations were carried out against all nine categories of promoter sequences in the test set (refer to Section 2.1). In addition, we also calculate the *Pearson Score* and *Spearman Score* metrics used in the competition to rank different submissions, which is a weighted average of performance across different categories of promoters in the test set. Overall, 20 different evaluation metrics were computed for each model.

#### 3.3.2 Qualitative, explanatory evaluation

Here, we assess Camformer in the context of model explainability, aiming to identify what the model is attending to when making its predictions. For this, we use the following two approaches (more details in Methods 1.4, available as supplementary data at *Bioinformatics Advances* online):

1. Gradient-weighted class activation maps (Grad-CAM): Grad-CAM (Selvaraju *et al.* 2017) highlights important regions in the input sequence by analysing the gradients of the model’s output with respect to the input to each convolutional layer.
2. *In silico* mutagenesis (ISM): ISM measures how the model responds to variations in the input sequence by systematically mutating each position of the input sequence and observing the change in the predicted output.

We further visualize the results using sequence logos, with an intention to highlight important TF motifs in the promoters. Further analyses of motif occurrences are described in Methods 1.5, available as supplementary data at *Bioinformatics Advances* online.

## 4 Results

### 4.1 Camformer robustly predicts gene expression

**Figure 3.**
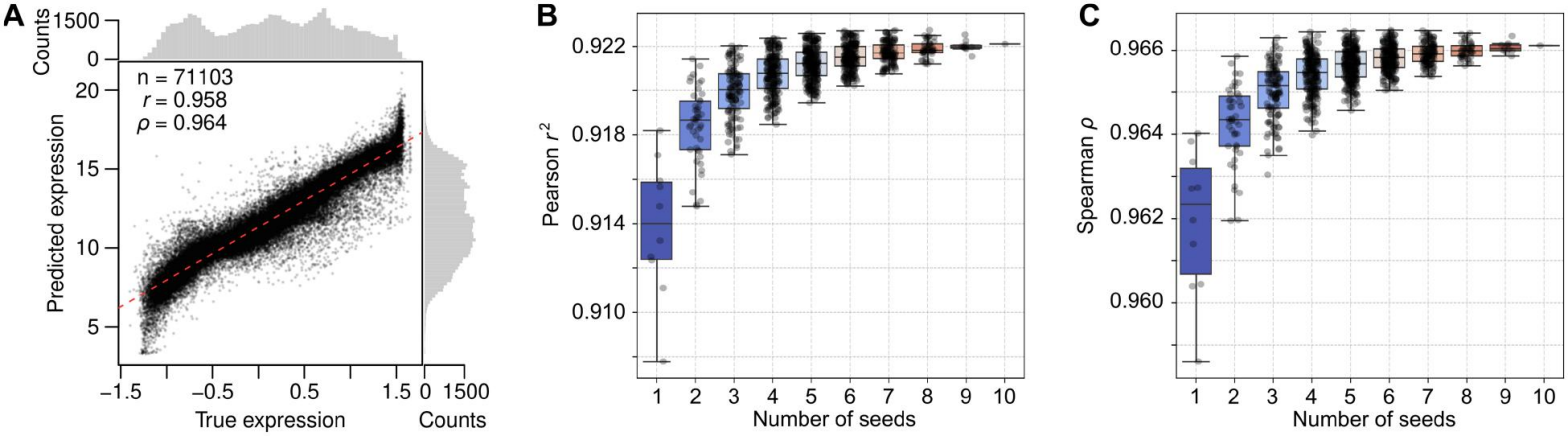
The model was evaluated on the DREAM2022 test data (*n* = 71 103). (A) Scatter plot between true and predicted expression values. (B) Predictive performance measured using Pearson $r^{2}$ across 10 replicate models and ensemble models constructed by taking the mean prediction from *k* replicates (where k∈{2,…,10}). A replicate here refers to a model constructed using a random seed. (C) Same as (B), but showing Spearman ρ.

Camformer placed fourth out of 110+ teams in the DREAM2022 challenge (Rafi *et al.* 2024). Notably, its reported performance ($r^{2}=0.913$, ρ=0.961) provided a substantial improvement over the reference model (Vaishnav *et al.* 2022) at 20th place ($r^{2}=0.879$, ρ=0.938). However, this evaluation did not assess the stability of model design and training strategy. We therefore re-trained the Camformer model 10 times using different random seeds. Out of the 10 models, more than half of them exceeded previously reported performance ($r^{2}=0.914\pm 0.003$, ρ=0.962±0.002, Fig. 3A–C, Fig. S2), showing that the model is not only accurate but also robust.

To investigate how model design influences its performance, we repeated the same experiment for Camformer-Mini, a small CNN (1.4M parameters) without residual connections, which was developed during the competition and later optimized (described below in Section 4.1.1). Although Camformer-Mini displayed lower performance ($r^{2}=0.898\pm 0.004$, ρ=0.952±0.003), it nevertheless exceeded previous SOTA (Fig. S3A–C).

A common strategy to improve prediction performance is to use an ensemble model, combining predictions from multiple models. Both Camformer and Camformer-Mini displayed significant performance gain and reduced variance when an ensemble was constructed by averaging predictions from several seeds (Fig. 3B and C, Fig. S3B and C). For instance, using two seeds significantly improved Camformer $r^{2}$ from 0.914±0.003 to 0.918±0.002 (*P *= .001, two-sided Student’s *t*-test) and ρ from 0.962±0.002 to 0.964±0.001 (*P *= .002). As expected, the best performance was observed using all 10 seeds ($r^{2}=0.922$, ρ=0.966). Thus, even without changing model design, performance can readily be improved by re-training the same model several times with different seeds.

#### 4.1.1 Optimizer and encoding contribute to performance

To find out what contributes to model performance, we performed a hyperparameter grid search. For this experiment, we considered Camformer (16.6 M parameters) and Camformer-Mini (1.4 M), and additionally a small variant of the Camformer, Camformer-Small (3.4 M), where the number of channels was reduced from 512 to 256 at each convolutional layer. In addition to model structure, we explored sequence encoding, loss function, optimizer, and LR scheduler (see Methods 1.3, available as supplementary data at *Bioinformatics Advances* online). In total, 90 hyperparameter combinations were evaluated for each model structure, resulting in 270 models.

Notably, the AdamW optimizer improved median *r* by 4.3% compared to Lion (*p *= 7e-24, two-sided Wilcoxon Rank Sum test, *n* = 145 model comparisons) as shown in Fig. S4A. We therefore analysed the two optimizers separately (Fig. S4B and C). Using AdamW, both Camformer and Camformer-Small performed significantly better than Camformer-Mini (median *r* increased by 0.4%–0.5%, P< 5e−6, n=45). Moreover, across the five sequence encodings tested, encodings without an uncertainty channel (‘onehot’, ‘onehotWithP’, and ‘onehotWithN’) consistently performed better than those with uncertainty (‘onehotWithInt’ and ‘onehotWithBoth’), displaying a median *r* increase by 0.5%–0.6% across all comparisons (P< 2e−5, n=27). The same trend was seen for Lion, although the gain was more variable (0.5%–2%). Full results for the grid search are available on our GitHub repository.

**Figure 4.**
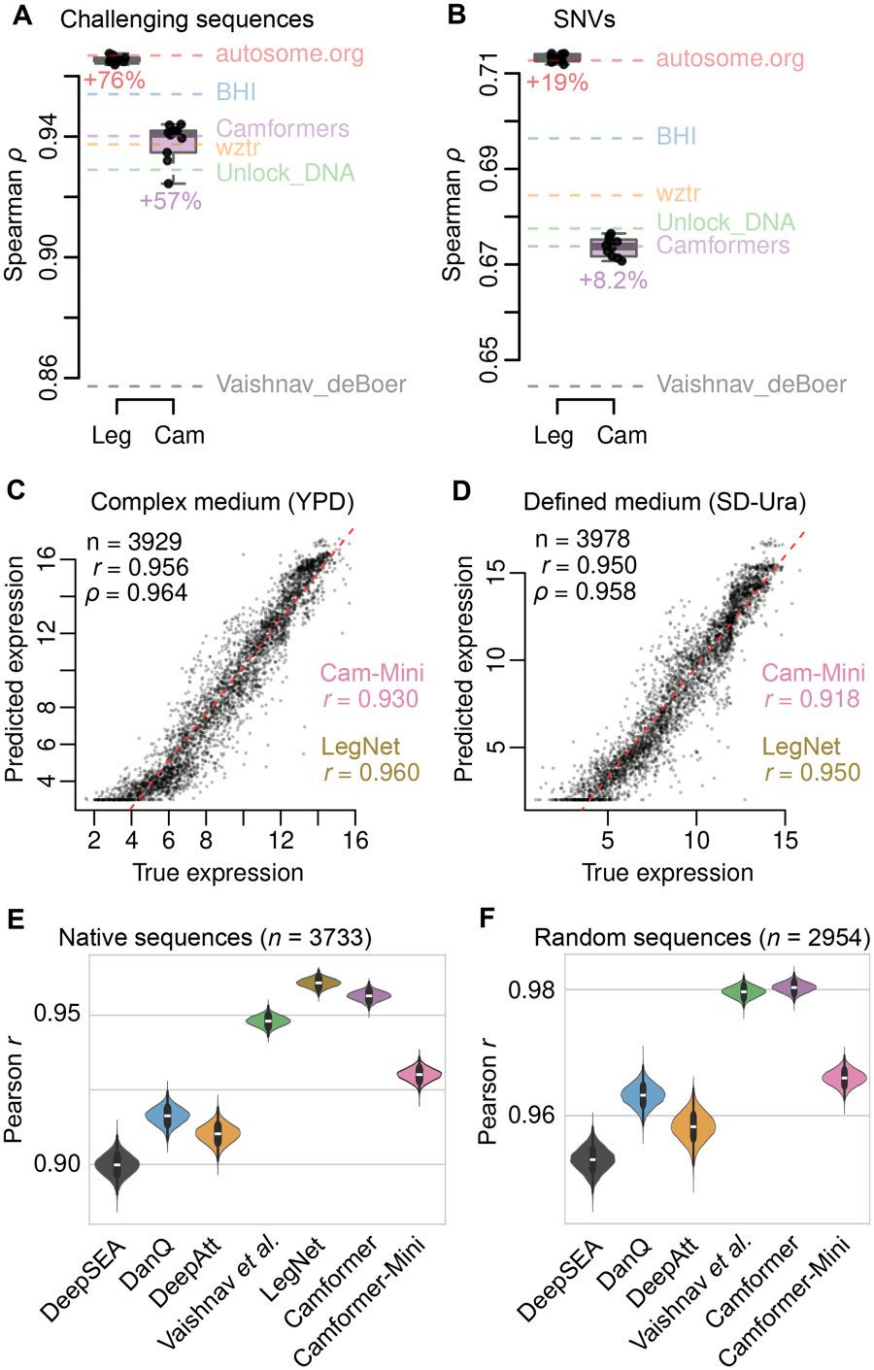
Evaluation and benchmark. (A, B) Camformer (Cam) and LegNet (Leg) performance across (A) challenging sequences, which displayed the strongest improvement over baseline (Vaishnav_deBoer; dashed grey line), or (B) SNVs, which displayed the weakest improvement over baseline. The top five DREAM2022 submissions are shown as coloured dashed lines. All sequence categories are shown in Figure S5. (C, D) Performance of Camformer in predicting expression for yeast grown in (C) complex or (D) defined medium. Performance of Camformer-Mini and LegNet is indicated. (E, F) Comparison of Camformer and Camformer-Mini with SOTA models. Prediction of expression for (E) native or (F) random promoters for yeast grown in complex medium. Violin plots show bootstraps with *n* = 10 000 replicates. All differences were significant (P<.05, paired Student’s *t*-test).

We next selected the best set of hyperparameters for each model structure and re-trained each model 10 times using all training data. Evaluating these models across the nine promoter categories in the test set demonstrated that the original Camformer model remained the best model (Figs S5–S7). We therefore used Camformer for the remaining analyses, unless otherwise stated.

**Figure 5.**
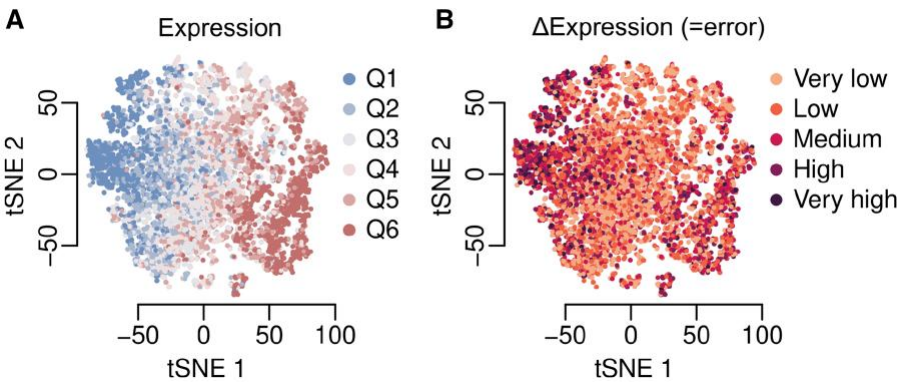
t-SNE embedding for 10 000 promoters from the test set. The scatter plots show the t-SNE embeddings of the activations in the last convolutional layer. (A) The points are coloured based on their expression quantiles, where Q1 is the lowest and Q6 is the highest. (B) The points are coloured based on their error quantiles from very low to very high error. Embeddings for all layers are shown in Fig. S9.

**Figure 6.**
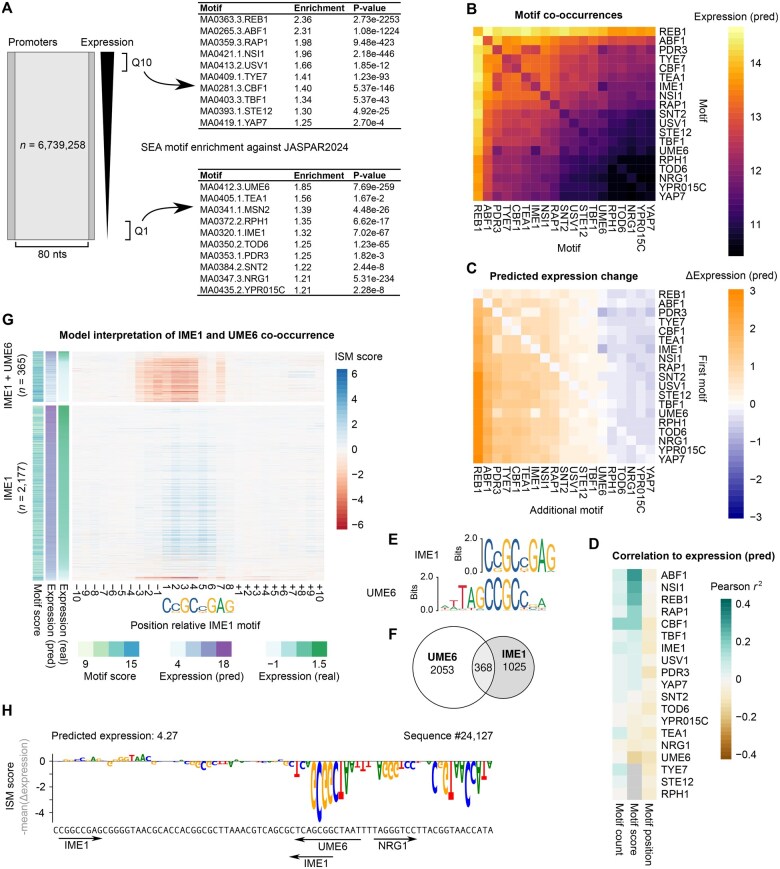
Camformer predictions reveal gene-regulatory logic. (A) The training data were divided into 10 expression quantiles (Q1 to Q10) and SEA was used to identify the most enriched motifs among the highest (Q10) and lowest (Q1) expressed promoters. (B) Heatmap showing mean predicted expression for training data sequences containing pairwise combinations of selected motifs as detected by FIMO. Baseline expressions for each individual motif are shown along the diagonal. (C) Heatmap showing mean expression difference between sequences with one motif (indicated by row) and sequences with two co-occurring motifs (row and column). (D) Heatmap showing correlation between predicted expression and motif count, score, or position. Grey values indicate cases where all motifs had the same score. (E) JASPAR2024 motif logos show that IME1 and UME6 motifs may overlap. (F) Venn diagram illustrating that some test set sequences had both motifs. (G) Heatmap showing ISM scores per position across IME1 motifs in the test set. The IME1 motifs are grouped into those that co-occur with UME6 (n=365) and those that appear on their own (n=2177). (H) Examples of motifs highlighted using ISM across variable positions 18–97. Positive values indicate contribution to higher expression and negative values indicate a contribution to lower expression. Positions and orientation of motifs are shown as arrows.

#### 4.1.2 Improvements on previously challenging sequences

To better understand its performance, we compared Camformer to a benchmark transformer model by Vaishnav *et al.* (2022). However, benchmark performance differed substantially across different sequence categories, ranging from $r^{2}=0.264$, ρ=0.305 for high expressed sequences to $r^{2}=0.912$, ρ=0.956 for random sequences (Figs S5 and S6). Since neither $r^{2}$ nor ρ can exceed 1, the maximum possible improvement therefore differs between difference categories.

To provide a fair comparison against different baselines, we therefore used baseline-normalized improvement, defined as $\Delta^{\mathrm{norm}}\rho =\Delta \rho /(1-\rho_{1})=(\rho_{2}-\rho_{1})/(1-\rho_{1})$. Normalized improvement in ρ was 36.9% for all sequences and ranged from 8.2% for SNVs, 13.5% for native, 29.5% for motif tiling, 30.3% for high expressed, 31.9% for low expressed, 36.1% for random sequences, 39.5% for motif perturbation, and 56.8% for challenging sequences (Fig. S5). Although normalized performance across most categories was increased around 30%–40%, the most notable exceptions were the strong improvement (56.8%) on challenging sequences (Fig. 4A) and the weak improvement (8.2%) on SNVs (Fig. 4B). Similarly, LegNet also achieved its lowest improvement at 19.3% for SNVs, potentially reflecting an inherent difficulty for a model trained on diverse random sequences to understand the meaning of SNVs (Fig. 4B).

#### 4.1.3 Predictive performance near SOTA

Vaishnav *et al.* (2022) previously released two additional datasets, representing yeast grown in complex (YPD) or defined (SD-Ura) media. Each dataset contains promoter sequences and their YFP expression measured using the same assay as the one used to train Camformer. The complex media data included 30 722 376 training and 3929 test sequences, whereas the defined media had 20 616 659 training and 3978 test sequences. In each case, the training data consisted of random sequences and the test sequences represented fragments from native yeast promoters. Despite these data representing yeast grown under different conditions, we first tested how Camformer and Camformer-Mini models performed on the two test sets. Notably, Camformer achieved strong performance (r=0.956, ρ=0.964 for complex media; r=0.950, ρ=0.958 for defined media) (Fig. 4C and D), similar to LegNet (r=0.960 and r=0.950 for complex and defined media, respectively).

We next re-trained Camformer and Camformer-Mini using the training data from the complex and defined media. To understand the relationship between training data size and model performance, we performed a subsampling experiment, using increasingly larger training data. For Camformer, we noticed a steady improvement with more training data. In contrast, Camformer-Mini displayed no clear improvement with additional training data, suggesting that Camformer-Mini may not be sufficiently parameterized (Fig. S8A and B).

Using Camformer and Camformer-Mini trained on complex media, we compared their performance to currently leading models, including DeepSEA, DanQ, DeepAtt, the benchmark model developed by Vaishnav *et al.* (2022), and LegNet, all re-trained using the same data (Fig. 4E and F). Notably, Camformer provided substantial improvement over DeepSEA, DanQ, DeepAtt, and the benchmark model, and was only marginally worse than LegNet for native yeast sequences (r=0.956), and displayed strong performance just above the benchmark also on random sequences (r=0.968). We conclude that the Camformer architecture provides SOTA performance also on very large training sets.

### 4.2 Sequence embeddings reveal decision process

We next studied the internal sequence embeddings learnt by Camformer. We randomly sampled 10 000 sequences from the test set and passed them on to the model. To visualize their sequence embeddings using tSNE, the (*h*, *w*, *c*)-dimensional output of each convolutional layer was flattened to a vector of length d=h×w×c. In Fig. S9, we show how the convolutional layers progressively group sequences of similar expressions together, with one of the strongest separations displayed by the sixth layer (Fig. 5A). Overall, residual errors were evenly distributed across the sequence space, although very lowly and very highly expressed sequences displayed slightly higher error rates (Fig. 5B). These results demonstrate that the model progressively extracts patterns in the promoter sequences that determine gene expression.

### 4.3 Camformer captures regulatory signals

Next, we asked whether associations can be learnt between promoter characteristics, such as TF binding site motifs, and gene expression. For this, we used two in silico approaches: (i) Gradient-weighted class activation map (Grad-CAM) and (ii) Input perturbation analysis through in silico mutagenesis (ISM).

#### 4.3.1 Grad-CAM highlights potential regulatory hierarchy

We first used Grad-CAM to determine what parts of each promoter sequence the model attends to while making predictions. In short, Grad-CAM estimates how a perturbation in the input sequence influences the predicted output. For this experiment, we applied Grad-CAM to all 71 103 sequences in the test set. Fig. S10 shows an example of a promoter with a strong motif that is found near the 3ʹ end across all convolutional layers, and two additional upstream motifs that are captured primarily in the deeper layers, potentially suggesting a hierarchy.

The average saliency map for all promoters is shown in Fig. S11. As expected, the model focuses on the variable positions 18 to 97. Moreover, the average maps indicate that the model focuses more towards the 3ʹ end of the sequences. This is consistent with a higher impact of TFs that bind near the core promoter and the transcription start site (TSS). Interestingly, the model was also strongly affected by GC content (Fig. S11). In agreement with this, we observed a strong correlation between GC content and expression (r=0.263, Fig. S12).

#### 4.3.2 ISM highlights TF motifs

While Grad-CAM and its variants remain among the most widely used tools for interpreting deep neural networks in genomics (Zhou *et al.* 2023), we further conducted an in silico saturation mutagenesis experiment. Here, we used sequences from the test set and systematically mutated each nucleotide across positions 18–97. This resulted in the creation of 240 new sequences—each with a different SNV—for each original sequence. We passed these to our model and recorded the predicted expression. Next, we quantified how each nucleotide in the original sequence contributes to gene expression as the negative mean per-nucleotide effect of each SNV. To find sequence motifs controlling gene expression, we calculated the entropy of the ISM sequence logo for each input promoter. Low entropy indicates high variation between nucleotide importance scores, and we hypothesized that such sequences would contain distinct regulatory motifs. Indeed, we observed that top-scoring sequences contained both activating and repressive elements, associated with high and low expression, respectively. Overall, there was a high concordance between ISM and Grad-CAM motifs, although motifs produced by ISM appears to be more well-defined (Fig. S13).

### 4.4 Model predictions reveal *cis*-regulatory logic

To demonstrate how Camformer can be used to infer *cis*-regulatory logic, we next performed a SEA enrichment analysis on the training data to identify motifs that are overrepresented in high- and low-expressed sequences, respectively (Fig. 6A). To reveal potential interactions, we calculated the mean expression of test set sequences for each combination of these motifs (Fig. 6B). While most individual motifs displayed only small differences in expression (Fig. 6B, diagonal), sequences containing the REB1 motif displayed highly elevated expression, consistent with its known role in displacing nucleosomes (Hartley and Madhani 2009). In contrast to individual motifs, many motif combinations resulted in substantially altered expression. To quantify this, we calculated the expression change between sequences with two motifs present together, compared to sequences containing only the first motif (Fig. 6C). This revealed that most motifs have a consistently activating (REB1, ABF1, PDR3, TYE7, CBF1, TEA1, IME1, NSI1, and RAP1) or repressive (YAP7, YPR015C, NRG1, TOD6, and RPH1) effect, when present together with any other motif. In contrast, UME6 had a strongly repressive effect, only in the presence of IME1 or PDR3. This may reflect competition between TFs, or an interaction between UME6 and IME1 (Harris and Ünal 2023).

Each detected motif has a score reflecting how well it matches the consensus motif. This score will roughly reflect its binding affinity and there should subsequently be a positive correlation between score and expression for activating motifs and a negative correlation for repressive motifs. Indeed, when exploring the correlation between predicted expression and motif count, score, and position (Fig. 6D), we observed a high correlation for the activator ABF1 and a low correlation for UME6. Additionally, having more motifs was generally weakly positively correlated with expression, and motif position negatively correlated, suggesting that motifs positioned too close to the TSS may inhibit efficient transcription.

We noticed that the interaction between UME6 and IME1 could partially be explained by motif similarities (Fig. 6E), with 386 sequences containing both motifs (Fig. 6F). To investigate whether our model could separate the effect of IME1 on its own, or together with UME6, we grouped the IME1 motifs by whether they overlapped a UME6 motif or not (Fig. 6G). Strikingly, the model consistently associated the presence of both motifs with a strong reduction in gene expression, as measured by the ISM score over the motif. In contrast, IME1 on its own had a neutral to weakly positive effect (Fig. 6G and H). These observations suggest that Camformer captures and can be used to study *cis*-regulatory logic.

## 5 Discussion

In this study, we present an in-depth analysis of Camformer, a CNN-based model developed to predict gene expression from promoter sequences. Despite recent advances in deep learning for genomics, Camformer demonstrates that it is possible to achieve SOTA performance by designing a model entirely from scratch, rather than building upon pre-existing blocks or architectures. One key advantage of this approach is the possibility to design small and bespoke models such as Camformer-Mini (1.4 M parameters), while maintaining competitive performance. In contrast, other top-performing models explored a range of recently developed architectures. For instance, LegNet used EfficientNetV2 (Tan and Le 2021) blocks, CRMnet used a transformer-encoded U-Net structure, and Proformer adopted a novel transformer architecture.

By thoroughly exploring the Camformer model design space, we show that the choice of sequence encoding and optimizer significantly impacts its performance. We systematically tested 270 model variants, but none outperformed the original model. However, using an ensemble approach, we demonstrate further improvement, as previously observed with LegNet (Penzar *et al.* 2023). This suggests that some residual errors could potentially be removed with a more refined model design.

Our work demonstrates that Camformer can be used to uncover *cis*-regulatory mechanisms underlying gene regulation. For instance, we show that Camformer recognizes the effect of key TF motifs, including REB1, AZF1, UME6, and YAP7. Moreover, our model captures an interaction or hierarchy between UME6 and IME1 motifs and can thus be used to study more complex *cis*-regulatory logic.

One difficulty in DREAM2022 was that while the training data consisted of random sequences, the test data sequences were not random, nor did they represent a comparable distribution of expression values. As a result, predicting gene expression on the test data was inherently difficult owing to a distribution shift (Wiles *et al.* 2022) between the training and test datasets. Consistent with this, all submitted models displayed strong performance on random sequences, as opposed to native sequences or sequences designed to drive unusually high or low expression levels (Rafi *et al.* 2024).

## Supplementary Material

vbaf130_Supplementary_Data

## Data Availability

The code repository for this study is available at https://github.com/Bornelov-lab/Camformer. The training and test data are available at https://zenodo.org/records/7395397.

## References

[vbaf130-B1] Akiba T , Sano S, Yanase T et al Optuna: a next-generation hyperparameter optimization framework. In: *Proceedings of the 25th ACM SIGKDD International Conference on Knowledge Discovery and Data Mining, Anchorage, AK*, pp. 2623–31. New York, NY, USA: Association for Computing Machinery, 2019.

[vbaf130-B2] Avsec Ž , Agarwal V, Visentin D et al Effective gene expression prediction from sequence by integrating long-range interactions. Nat Methods 2021b;18:1196–203. 34608324 10.1038/s41592-021-01252-x PMC8490152

[vbaf130-B3] Avsec Ž , Weilert M, Shrikumar A et al Base-resolution models of transcription-factor binding reveal soft motif syntax. Nat Genet 2021a;53:354–66. 33603233 10.1038/s41588-021-00782-6 PMC8812996

[vbaf130-B4] de Boer CG , Vaishnav ED, Sadeh R et al Deciphering eukaryotic gene-regulatory logic with 100 million random promoters. Nat Biotechnol 2020;38:56–65. 31792407 10.1038/s41587-019-0315-8 PMC6954276

[vbaf130-B5] Ding K , Dixit G, Parker BJ et al CRMnet: a deep learning model for predicting gene expression from large regulatory sequence datasets. Front Big Data 2023;6:1113402. 36999047 10.3389/fdata.2023.1113402 PMC10043243

[vbaf130-B6] Gerstein MB , Kundaje A, Hariharan M et al Architecture of the human regulatory network derived from encode data. Nature 2012;489:91–100. 22955619 10.1038/nature11245 PMC4154057

[vbaf130-B7] Guo Y , Mahony S, Gifford DK. High resolution genome wide binding event finding and motif discovery reveals transcription factor spatial binding constraints. PLoS Comput Biol 2012;8:e1002638. 22912568 10.1371/journal.pcbi.1002638 PMC3415389

[vbaf130-B8] Harris A , Ünal E. The transcriptional regulator ume6 is a major driver of early gene expression during gametogenesis. Genetics 2023;225:iyad123. 37431893 10.1093/genetics/iyad123 PMC10550318

[vbaf130-B9] Hartley PD , Madhani HD. Mechanisms that specify promoter nucleosome location and identity. Cell 2009;137:445–58. 19410542 10.1016/j.cell.2009.02.043 PMC2677553

[vbaf130-B10] Jumper J , Evans R, Pritzel A et al Highly accurate protein structure prediction with AlphaFold. Nature 2021;596:583–9. 34265844 10.1038/s41586-021-03819-2 PMC8371605

[vbaf130-B11] Kwak I-Y , Kim B-C, Lee J et al Proformer: a hybrid macaron transformer model predicts expression values from promoter sequences. BMC Bioinformatics 2024;25:81. 38378442 10.1186/s12859-024-05645-5 PMC10877777

[vbaf130-B12] Li J , Pu Y, Tang J et al DeepATT: a hybrid category attention neural network for identifying functional effects of DNA sequences. Brief Bioinform 2020;22:bbaa159. 10.1093/bib/bbaa159 32778871

[vbaf130-B13] Lin T , Wang Y, Liu X et al A survey of transformers. AI Open 2022;3:111–32.

[vbaf130-B14] Meyer P , Saez-Rodriguez J. Advances in systems biology modeling: 10 years of crowdsourcing dream challenges. Cell Syst 2021;12:636–53. 34139170 10.1016/j.cels.2021.05.015

[vbaf130-B15] Penzar D , Nogina D, Noskova E et al LegNet: a best-in-class deep learning model for short DNA regulatory regions. Bioinformatics 2023;39:btad457. 37490428 10.1093/bioinformatics/btad457 PMC10400376

[vbaf130-B16] Quang D , Xie X. DanQ: a hybrid convolutional and recurrent deep neural network for quantifying the function of DNA sequences. Nucleic Acids Res 2016;44:e107. 27084946 10.1093/nar/gkw226 PMC4914104

[vbaf130-B17] Rafi AM , Nogina D, Penzar D et al A community effort to optimize sequence-based deep learning models of gene regulation. Nat Biotechnol 2024. 10.1038/s41587-024-02414-w PMC12339383 39394483

[vbaf130-B18] Selvaraju RR , Cogswell M, Das A et al Grad-CAM: visual explanations from deep networks via gradient-based localization. In: *Proceedings of IEEE International Conference on Computer Vision, Venice, Italy*, pp. 618–26. 2017.

[vbaf130-B19] Tan M , Le QV. Efficientnetv2: Smaller models and faster training. In: *Proceedings of the 38th International Conference of Machine Learning, Virtual*, pp. 10096–106. 2021.

[vbaf130-B20] Vaishnav ED , de Boer CG, Molinet J et al The evolution, evolvability and engineering of gene regulatory DNA. Nature 2022;603:455–63. 35264797 10.1038/s41586-022-04506-6 PMC8934302

[vbaf130-B21] Wiles O, Gowal S, Stimberg F et al A fine-grained analysis on distribution shift. In: *International Conference on Learning Representations, Virtual*. 2022.

[vbaf130-B22] Zhou J , Troyanskaya OG. Predicting effects of noncoding variants with deep learning–based sequence model. Nat Methods 2015;12:931–4. 26301843 10.1038/nmeth.3547 PMC4768299

[vbaf130-B23] Zhou Z, Hu M, Salcedo M et al XAI meets biology: a comprehensive review of explainable ai in bioinformatics applications. arXiv, arXiv:2312.06082, 2023, preprint: not peer reviewed.

[vbaf130-B24] Zrimec J , Börlin CS, Buric F et al Deep learning suggests that gene expression is encoded in all parts of a co-evolving interacting gene regulatory structure. Nat Commun 2020;11:6141. 33262328 10.1038/s41467-020-19921-4 PMC7708451

